# I Want to but I Won't: Pluralistic Ignorance Inhibits Intentions to Take Paternity Leave in Japan

**DOI:** 10.3389/fpsyg.2017.01508

**Published:** 2017-09-20

**Authors:** Takeru Miyajima, Hiroyuki Yamaguchi

**Affiliations:** ^1^Graduate School of Human-Environment Studies, Kyushu University Fukuoka, Japan; ^2^Faculty of Human-Environment Studies, Kyushu University Fukuoka, Japan

**Keywords:** pluralistic ignorance, paternity leave in Japan, norm perpetuation, gender role norm, conservative lag

## Abstract

The number of male employees who take paternity leave in Japan has been low in past decades. However, the majority of male employees actually wish to take paternity leave if they were to have a child. Previous studies have demonstrated that the organizational climate in workplaces is the major determinant of male employees' use of family-friendly policies, because males are often stigmatized and fear receiving negative evaluation from others. While such normative pressure might be derived from prevailing social practices relevant to people's expectation of social roles (e.g., “Men make houses, women make homes”), these social practices are often perpetuated even after the majority of group members have ceased to support them. The perpetuation of this unpopular norm could be caused by the social psychological phenomenon of pluralistic ignorance. While researches have explored people's beliefs about gender roles from various perspectives, profound understanding of these beliefs regarding gender role norms, and the accuracy of others' beliefs remains to be attained. The current research examined the association between pluralistic ignorance and the perpetually low rates of taking paternity leave in Japan. Specifically, Study 1 (*n* = 299) examined Japanese male employees' (ages ranging from the 20 s to the 40 s) attitudes toward paternity leave and to estimate attitudes of other men of the same age, as well as behavioral intentions (i.e., desire and willingness) to take paternity leave if they had a child in the future. The results demonstrated that male employees overestimated other men's negative attitudes toward paternity leave. Moreover, those who had positive attitudes toward taking leave and attributed negative attitudes to others were less willing to take paternity leave than were those who had positive attitudes and believed others shared those attitudes, although there was no significant difference between their desires to take paternity leave. Study 2 (*n* = 425) replicated these results and further indicated that they could not be explained by the participants' needs to be socially desirable. Together, our findings suggest that pluralistic ignorance occurs in the context of taking paternity leave in Japanese men, and this leads to the low use of available paternity leave.

## Introduction

*Paternity leave* refers to a father's taking a temporary leave of absence from work to care for a newborn. Parental roles have traditionally been gendered in families with young children; that is, men have been primarily responsible for earning and women for caring. However, most OECD countries today provide statutory paternity leave to encourage gender equality between parents (Hegewisch and Gornick, 2011; Mun and Brinton, 2015). Unlike most developed countries, where the use of paternity leave has become commonplace, the rate of paternity leave utilization in Japan has never exceeded 3% (Japanese Cabinet Office, 2015). This is despite the fact that in recent decades the number of dual-income households (i.e., both husband and wife work) greatly exceeded that of households with full-time homemakers (i.e., only one spouse, usually the husband, works; Japanese Cabinet Office, 2014). This suggests that in Japan, mothers are still more likely to be responsible for childcare. In line with this argument, the Japanese women's labor force participation is characterized as an M-shaped curve: The majority leave employment at the time of marriage or first birth to take care of their children, and might return to work after their children are old enough (National Institute of Population Social Security Research, 2015).

This inequality in childcare responsibilities might undermine the empowerment of women and be harmful for men as well. That is, the family role as “caregiver” could often interfere with work roles, and work role as a “breadwinner” could also interfere with family roles. Previous studies have revealed that incompatible demands between work and family roles (i.e., work–family conflict) are associated with physical and mental health problems as well as job-related outcome (e.g., job satisfaction, employee turnover, and absenteeism; Allen et al., 2000; Aryee et al., 2005; McNall et al., 2010; Amstad et al., 2011; Butts et al., 2013). The Japanese government has addressed the country's perpetually low rate of paternity leave utilization. For instance, it conducts publicity activities to increase men's awareness, holds a workshop for managers to encourage the use of paternity leave by their male employees, and provides corrective guidance if men are harassed for using paternity leave (Japanese Cabinet Office, 2015). Despite a number of governmental intervention strategies designed to improve the use of paternity leave, these problems remain.

One possible explanation for the low rate of taking paternity leave among Japanese men is that they still cling to traditional gender-role norms, expressed as “*Men make houses, women make homes*.” Indeed, the vast majority of Japanese supported this norm in the 1980s. However, the norm had lost widespread support by 2002 (Japanese Cabinet Office, 2002). In accord with this change in people's values, the Japanese government established the Child Care and Family Care Leave Act in 1991 to encourage work–family balance. The law secures the rights of both men and women to take parental leave, and prevents unfair treatment by employers of those who use such leave. A recent opinion poll showed that over 60% of Japanese men from their 20 to 40 s aspired to take paternity leave if they were to have a child (NetMile Research, 2009). That is, the stereotypical gender roles do not seem to be as deeply rooted as they once were. Nevertheless, men's use of paternity leave has remained at a low level over the past several decades. Taken together, these findings suggest that the persistence of traditional gender-role norms is not the primary determinant of the low rate of taking paternity leave.

Another possible explanation for male employees' passivity with regard to requesting parental leave is perceived hostility in their workplaces toward taking leave (Blair-Loy and Wharton, 2002; Japanese Ministry of Health, 2012; Coltrane et al., 2013). If male employees behave against their gender stereotype (i.e., requesting paternity leave to be involved as caregivers of children), they often suffer from workplace harassment (Berdahl and Moon, 2013; Rudman and Mescher, 2013). For instance, fathers who have requested family leave have received lower evaluations of their work (Judiesch and Lyness, 1999; Vandello et al., 2013) and organizational commitment (Allen and Russell, 1999), and suffered more negative reactions from evaluators than have women (Wayne and Cordeiro, 2003). Consequently, male employees tend to be reluctant to take advantage of family-friendly policies because of the fear of negative evaluation from their colleagues and superiors in the workplace (Blair-Loy and Wharton, 2002; Ueda and Kurosawa, 2012; Vandello et al., 2013). This hostile organizational climate toward counter-stereotypical behavior might derive from prevailing social practices relevant to people's expectations of social roles. Specifically, when people behave in a gender-counter-stereotypical way, they are likely to be evaluated unfavorably (Rudman, 1998; Heilman and Wallen, 2010; Moss-Racusin et al., 2010; i.e., backlash effects). This negative reaction could be explained by social role theory (Eagly, 1987), which proposes that perceived incongruence between gender stereotype and social behavior leads to prejudice.

While prevailing social expectations of gender role may shape our beliefs and behaviors, these social expectations might change over time. For instance, recent research (Lopez-Zafra and Garcia-Retamero, 2012, Spanish sample; Wilde and Diekman, 2005, US and German sample; Yukawa and Hirooka, 2003, Japanese sample) has examined beliefs about men and women (i.e., masculine-feminine characteristics) in the past, present, and future. The results revealed that participants perceived decreasing differences between genders. Therefore, existing dominant social norms could change with time. Here, assuming that the majority of men now have positive attitudes toward paternity leave, how and why do they perceive an anti-paternity leave climate, and who harasses the counter-stereotypical fathers in their workplaces? Importantly, some social practices continue to be perpetuated even after the majority of group members have ceased to support them. The perpetuation of unpopular norms could be caused by the social psychological phenomenon termed *pluralistic ignorance*.

The term “pluralistic ignorance” was coined to describe the situation in which almost all members of a group privately reject group norms, yet believe that virtually all other group members accept them (Katz and Allport, 1931). Under such situations, individuals predict that they would lose social standing if they behaved as they wished. Behaving against the group norm could result in negative reactions from other ingroup members. Therefore, people are likely to follow perceived group norms to maintain positive impressions in their groups, even when they do not support the norms (Miller and McFarland, 1987; Miller and Prentice, 1994; Prentice and Miller, 1996; Geiger and Swim, 2016). In line with this idea, in situations of pluralistic ignorance, some people even actively enforce the perceived norms (i.e., publicly criticizing a “misfit” into accepting the norm), although they privately disapprove of the norms (Willer et al., 2009). Consequently, public behaviors of groups as a whole do not coincide with the majority of group members' private preferences under circumstances of pluralistic ignorance. Thus, the situation of pluralistic ignorance is well represented in the following sentence: “No one believes, but everyone believes that everyone else believes” (Krech and Crutchfield, 1948).

Previous researchers have examined pluralistic ignorance across many topics, including heavy drinking (college students estimated that other students felt more comfortable drinking heavily than they did; Prentice and Miller, 1993); racial segregation in the late 1960s and early 1970s (segregation had lost its support from most white Americans, but most white Americans believed that most others still supported it; Fields and Schuman, 1976); casual sex (students believed that their peers felt more comfortable engaging in unsafe sexual activity than they did; Lambert et al., 2003), bullying (children believed that their classmates approved of bullying more than they did; Sandstrom et al., 2013); adolescent delinquency (adolescents believed that their friends approved of delinquency more than they did; Young and Weerman, 2013) and flexibility bias (people estimated that others were more likely to stigmatize an employee who used a flexible work option; Munsch et al., 2014). Two common patterns are evident in the aforementioned findings: many people comply with unreasonable values without thinking carefully about the worthiness of the values, because of longstanding familiarity (Miller and Prentice, 1994); and public behavior is guided by perceived norms because of fear of losing social acceptance (Prentice and Miller, 1996). These characteristics correspond to the current situation with regard to paternity leave in Japan. Therefore, pluralistic ignorance is possibly involved with the perpetually low rate of using paternity leave in Japan. We tested this prediction.

Although studies have explored people's beliefs about gender roles from various perspectives, profound understanding of these beliefs with regard to gender role norms and the accuracy of others' beliefs remains to be developed. Elucidating a detailed mechanism of the issue could contribute to effective promotion of the use of paternity leave. Fathers' use of paternity leave might lead to benefits for themselves, their families and their workplaces. At the individual level, taking paternity leave increases fathers' involvement with childcare (Tanaka and Waldfogel, 2007, UK sample) and improves mental wellbeing (Strandh, 2000, Swedish sample), and is positively associated with their perceptions of advancement in organizations (King et al., 2009, U.S. sample). Moreover, fathers actively engaged in raising their children can benefit the children's development (Johnson et al., 2013, Australian sample; Pougnet et al., 2011, Canadian sample). At the organizational level, it has been suggested that provision of paternity leave enhances organizational commitment (Giffords, 2009, U.S. sample), employee satisfaction with the workplace environment, and productivity (Grover and Crooker, 1995, U.S. sample).

The present study investigated the association between pluralistic ignorance and the perpetually low rates of taking paternity leave in Japan. We sought to provide further insight into the perpetually low rates of utilizing paternity leave by examining whether male employees perceived the social norm accurately, and whether their behavioral intentions would be influenced by the perceived norm. To date, no empirical studies have directly examined the association between pluralistic ignorance and this social issue in Japan. On the basis of the previously noted studies on pluralistic ignorance and paternity leave, we hypothesized that male employees overestimate other men's negative attitudes toward paternity leave, and that this misperception inhibits the behavioral intention to take leave. To test these hypotheses, we conducted a web-based questionnaire with the panels of a crowdsourcing service to collect a large sample of adults who might be concerned in the current social issue. We measured participants' own private attitudes and estimations of other men's attitudes toward paternity leave. Furthermore, we examined the effect of misperceptions on behavioral intentions to take paternity leave.

*Hypothesis 1*: Male employees misperceive other male employees' attitudes toward paternity leave as significantly more negative than these attitudes actually are.

*Hypothesis 2*: This misperception inhibits the behavioral intention to take paternity leave, despite the aspiration to take such leave.

## Ethics statement

In accordance with the Declaration of Helsinki, the study was approved by the research ethics committee of the Faculty of Human-Environment Studies at Kyushu University (approval number 2016-016). We provided the aim of the survey on the first page, and asked participants to proceed to the subsequent survey only if they agreed with the instructions. Therefore, answering the survey was taken as agreement with the instructions and as assent to participate. All data were collected anonymously.

## Study 1

The aim of Study 1 was to directly examine the association between pluralistic ignorance and the perpetually low rates of taking paternity leaves in Japan.

### Materials and methods

#### Participants

Participants were recruited through the Yahoo! Crowdsourcing service (http://crowdsourcing.yahoo.co.jp). We aimed to examine the discrepancy between private attitudes and estimation of others' attitude toward paternity leave, as well as attitudes' influence on behavioral intentions to use leave. For this reason, people in their 50 and 60 s were excluded because they are less likely to have children in the future. Asking single men how they would behave when they have children may be impractical. Furthermore, it may be conceivable that men do not take paternity leave because they assume it is not available at their workplaces. Therefore, the selected inclusion criteria deemed critical for providing an adequately rigorous examination of the hypotheses were the following: (a) Japanese male between 20 and 49 years of age, (b) married, and (c) currently working in a workplace where a parental leave system is available for male employees. We publicized the survey page disclosing selection criteria, compensation for participation and estimated time required to complete within the Yahoo! Crowdsourcing panel service.

Participants completed an online questionnaire on paternity leave in exchange for a 4.25 JPY cash payment in April 2016. The questionnaire included an attention check question developed to detect satisficing in survey responses (Oppenheimer et al., 2009). Because this item (“Please choose option 1 for this item.”) has a clearly correct answer, choosing a non-correct option means that the participant has not carefully attended to the questionnaire. To ensure sufficient quality of data, participants who responded incorrectly to this item were eliminated from the analysis, leaving a total of 299 participants in the final analysis (*M*_age_ = 39.2, *SD* = 5.55, Range = 20–49). Power analysis revealed that this sample size could provide a power of 0.80 to detect medium-sized effects regarding the discrepancy between private attitude and estimation of others' attitudes (minimum *N* = 128), as well as attitudes' influence on behavioral intentions (minimum *N* = 211). Of the 299 participants, 98.3% identified as regular employees, with mean employment time in the current workplace 13.15 years (*SD* = 0.97). The average number of children was 1.62 (*SD* = 0.97).

#### Measures

##### Attitudes toward paternity leave

The participants' own attitudes toward paternity leave were measured by using five questions with a range of response options (“*To what extent do you have a positive attitude toward paternity leave?” [1. very negative to 6. very positive]*; “*How much would you approve of a man who takes paternity leave?” [1. I would not approve of him at all. to 6. I would approve of him strongly.]*; *How much would you support a man who takes paternity leave? [1. I would not support him at all. to 6. I would support him strongly.]*; *How highly would you evaluate a man who takes paternity leave? [1. I would evaluate him quite unfavorably. to 6. I would evaluate him quite favorably.]*; *How likable would you find a man who takes paternity leave? [1. I would find him not likable at all. to 6. I would find him very likable.]*). The scale achieved a Cronbach's alpha of 0.94 and McDonald's omega (Dunn et al., 2014) of 0.94, 95% CI [0.92, 0.95]. At the same time, their perceptions of others' attitudes were measured (e.g., “*To what extent do men in their 20s to 40s have positive attitudes toward paternity leave?” [1. very negative to 6. very positive]*; “*How much would men in their 20s to 40s approve of a man who takes paternity leave?” [1. They would not approve of him at all. to 6. They would approve of him strongly.]*). The scale achieved a Cronbach's alpha of 0.95 and McDonald's omega of 0.95, 95% CI [0.93, 0.96]. Items were created for the current study.

Previous studies have sought to capture pluralistic ignorance by comparing private attitudes and perceived social norms (e.g., Prentice and Miller, 1993). Therefore, in the current study, participants were asked to indicate their own attitudes toward paternity leave and to estimate the average attitudes of men aged 20–49 years. The aim was to measure discrepancies between private attitudes (i.e., actual preferences) and estimation of others' attitudes (i.e., perceived social norms) on paternity leave.

We computed the average across all private attitudes to create a “private attitude” score, and an average across all perceptions of others' attitudes to create an “estimation of others” attitudes' score. In both cases, higher scores indicate more positive attitudes toward paternity leave. The average scores were normally distributed (private attitudes: skewness = −0.22, kurtosis = −0.59; perceptions of others' attitudes: skewness = 0.27, kurtosis = −0.03), suggesting that parametric methods would be appropriate for data analysis.

##### Behavioral intention to take paternity leave

Next, participants were asked to indicate how likely they would be to take paternity leave if they had a child in the future. They were asked to consider both their *desire* and *willingness* to take leave, which aimed to identify inconsistencies between their behavioral preferences and behavioral intentions in a future situation. These items were created for the current study. The participants were asked the following question to determine their desire to take paternity leave: “*Would you want to take paternity leave if you had a child in the future?”* Response options were on a 7-point Likert scale (*1* = *I would never want to take it, 4* = *neutral, 7* = *I would definitely want to take it*).

The following item determined willingness to take paternity leave: “*How likely would you be to take paternity leave if you had a child in the future?*” This item also used a 7-point Likert scale (*1* = *I would never take it, 4* = *neutral, 7* = *I would definitely take it*). Both responses were confirmed to be normally distributed (desire to take paternity leave: skewness = −0.17, kurtosis = −0.82; willingness to take paternity leave: skewness = 0.60, kurtosis = −0.48).

##### Control variables

*Traditional gender role orientation*. The participants were asked to rate the extent to which they agreed to the following statements: *Husbands should work outside the home, and wives should take care of their families; Parenting is the most important career for women; A woman belongs in the home, and a man belongs in the workplace; Men are expected to earn income, and women are expected to take care of the family*. A 7-point Likert scale was used (*1. strongly disagree to 7. strongly agree*)^1^. These four items were created by the authors based on the reversed items of the short form of the Scale of Egalitarian Sex Role Attitudes (SESRA-S; Suzuki, 1994), used to measure participants' traditional gender role orientation. Since previous studies have shown that the more traditional gender-role oriented individuals are, the more strongly they adhere to gender-typed behaviors (Orlofsky et al., 1985), we applied this measurement to control this potential effect on attitudes and behavioral intentions to take paternity leave. The Cronbach's alpha was 0.89 and McDonald's omega of 0.89, 95% CI [0.87, 0.91]. Higher scores indicated more traditional gender role attitudes. In subsequent analysis, we used average scores of the four items.

*Demographic variables*. We also collected participants' age, number of children, length of employment service, and employment status. Previous studies indicated that the father's age, the number of children (Salmi et al., 2014, Finnish sample) and employment status (Brandth and Kvande, 2002, Norwegian sample) are associated with use of paternity leave. The authors assumed that longer length of service (i.e., job tenure) might enhance one's willingness to speak up for their statutory right to take paternity leave, because employees with relatively high organizational status are more likely to express dissenting opinions to supervisors (Kassing and Armstrong, 2001). These control variables were included in the model to provide a more exacting inspection of the hypotheses.

##### Operational definition of pluralistic ignorance and analytic plan

Previous studies have considered “perceived discrepancy score” as an operational definition of pluralistic ignorance. This score is calculated by subtracting the private attitude score from the others' attitudes score (e.g., Vandello et al., 2009; LaBrie et al., 2010; Sandstrom et al., 2013). High discrepancy scores represent overestimation of others' negative attitudes; low scores represent underestimation of these attitudes; and zero scores represent accurate estimation of attitudes. For instance, Sandstrom et al. (2013) examined the association between discrepancy score and behavioral response in the context of witnessed bullying among elementary school students. A high discrepancy score was positively correlated with participation in bullying (i.e., starting bullying, helping the bully, or laughing at victims of bullying). A high discrepancy score was negatively correlated with actively defending victims (i.e., trying to make the offender stop bullying). Thus, a discrepancy between one's own attitudes and estimation of others' attitudes inhibits individuals from freely exercising their behavioral preferences.

Although the aforementioned technique has been used in the literature, there is a scoring issue; namely, individuals who report their private attitudes on the relevant topic as “*6. strongly positive”* and estimated others' attitudes as “*4. slightly positive*,” cannot be distinguished from those who report their attitudes as “*3. slightly negative”* and estimated others' attitudes as “*1. strongly negative”* (i.e., both individuals' scores would be -2.). In other words, relative magnitude differences cannot be distinguished from attitude mismatches. Because pluralistic ignorance represents, by definition, a mismatch in perception of one's own and other's attitudes (Katz and Allport, 1931), the phenomenon should be analyzed in terms of such divergence, rather than relative magnitude differences.

To address this issue, in the subsequent analysis we divided participants into four categories according to their attitudes toward paternity leave and their estimations of others' attitudes. This procedure allowed us to examine whether pluralistic ignorance is associated directly with individuals' restricting their behavioral preferences. Moreover, this categorization technique is not susceptible to the relative magnitude problem because it enables analysis of the relationship between self-other discrepancy and behaviors based on individuals' absolute divergence of attitudes of self and others. The cut-off value for categorization was 3.5. In this study, both private attitude and estimation of others' attitudes were measured with five items on a 6-point Likert scale. The variables were calculated by averaging these items. In this case, the midpoint of the average score would be classified as either 3.4 or 3.6, but never 3.5. That is, participants' attitudes were always classified as either positive (i.e., 3.5 < average score) or negative (i.e., 3.5 > average score). We labeled participants who responded that other men felt negatively about paternity leave even though they themselves felt positively about it (self–other discrepancy group; *N* = 118)^2^ as victims of pluralistic ignorance. The categorization was implemented further as follows: Participants with positive attitudes toward paternity leave (average score > 3.5 on the 6-point Likert scale) who estimated that others would respond in the same manner as themselves (average score > 3.5) constituted the “positive attitudes” group (*N* = 103). Participants with negative attitudes toward paternity leave (average score < 3.5) who estimated that others had positive attitudes toward paternity leave (average score > 3.5) were excluded from the analysis because this group was considered too small (*N* = 9) to provide sufficient data. Finally, participants with negative attitudes toward paternity leave (averaged score < 3.5) who estimated that others would respond in the same manner as themselves (averaged score < 3.5) constituted the negative attitudes group (*N* = 69).

### Results

The means, standard deviations and correlations of the variables are listed in Table 1.

**Table 1:** Descriptive statistics and zero-order correlations in Study 1.

	***M***	***SD***	**1**	**2**	**3**	**4**	**5**	**6**	**7**	**8**	**9**
1. Age	39.20	5.55	–								
2. Number of children	1.62	0.97	0.23^**^	−							
3. Employment status	–	–	−0.01	−0.06	−						
4. Length of service	13.15	7.59	0.60^**^	0.18^**^	−0.16^**^	–					
5. Traditional gender role orientation	3.41	1.42	0.14^*^	0.06	0.05	0.14^*^	–				
6. Private attitudes	4.22	1.23	−0.16^**^	0.01	0.01	−0.14^*^	−0.46^**^	–			
7. Estimation of others' attitudes	3.16	1.00	−0.08	0.01	0.00	−0.06	−0.10^+^	0.37^**^	–		
8. Behavioral intention (desire)	4.31	1.73	−0.14^*^	−0.02	−0.05	−0.13^*^	−0.33^**^	0.74^**^	0.30^**^	–	
9. Behavioral intention (willingness)	3.24	1.71	−0.01	0.01	−0.03	−0.07	−0.25^**^	0.53^**^	0.42^**^	0.65^**^	–

*N = 299; employment status (1 = regular employee, 2 = contract employee)*.

** *p < 0.01*,

* *p < 0.05*,

+ *p < 0.10*.

#### Occurrence of pluralistic ignorance

To assess the self–other discrepancy in attitudes toward paternity leave, we conducted analysis of covariance, including age, number of children, length of service, employment status, and traditional gender role orientation in the model as covariates. More specifically, private attitude and estimation of others' attitudes were compared using within-subjects factorial design. (In the current study, the aforementioned demographic variables were controlled as extraneous variables.) This is a common technique for measuring pluralistic ignorance in the literature, and a significant difference between the means of private attitudes and estimations of others' attitudes has been considered the defining feature of the phenomenon (Perkins and Berkowitz, 1986; Miller and McFarland, 1987; Prentice and Miller, 1993).

Traditional gender role orientation was significantly linked to attitudes [*F*_(1, 292)_ = 39.50, *p* < 0.001, η_p_^2^ = 0.12]. As depicted in Table 1, traditional gender role orientation was significantly correlated with private attitude. While a higher score on traditional gender role orientation indicated a conservative attitude toward gender role, a higher score on private attitude toward paternity leave indicated a high gender-egalitarian orientation. Therefore, these results suggest that traditional gender role orientation could partially explain the variance of private attitude toward paternity leave. None of the effects of covariates except traditional gender role orientation were significant (*p*s > 0.19). The results showed that estimations of others' attitudes toward paternity leave (*M* = 3.16, *SD* = 1.00) were significantly lower than were participants' actual attitudes [*M* = 4.22, *SD* = 1.22; *F*_(1, 297)_ = 212.25, *p* < 0.001, η_p_^2^ = 0.42]. That is, participants estimated the attitudes of other men of the same age toward paternity leave to be more negative than they actually were; therefore, Hypothesis 1 was supported. Furthermore, the result confirmed that the validity of the categorization rule was sufficient, which demonstrated that private attitudes and the estimations of others' attitudes significantly diverged across the midpoint [private attitude: *t*_(292)_ = 10.15, *p* < 0.001, *d* = 0.88; estimation of others' attitudes: *t*_(292)_ = −5.83, *p* < 0.001, *d* = 0.47].

#### Influence of perceived self–other discrepancy on behavioral intention

**Figure 1:**
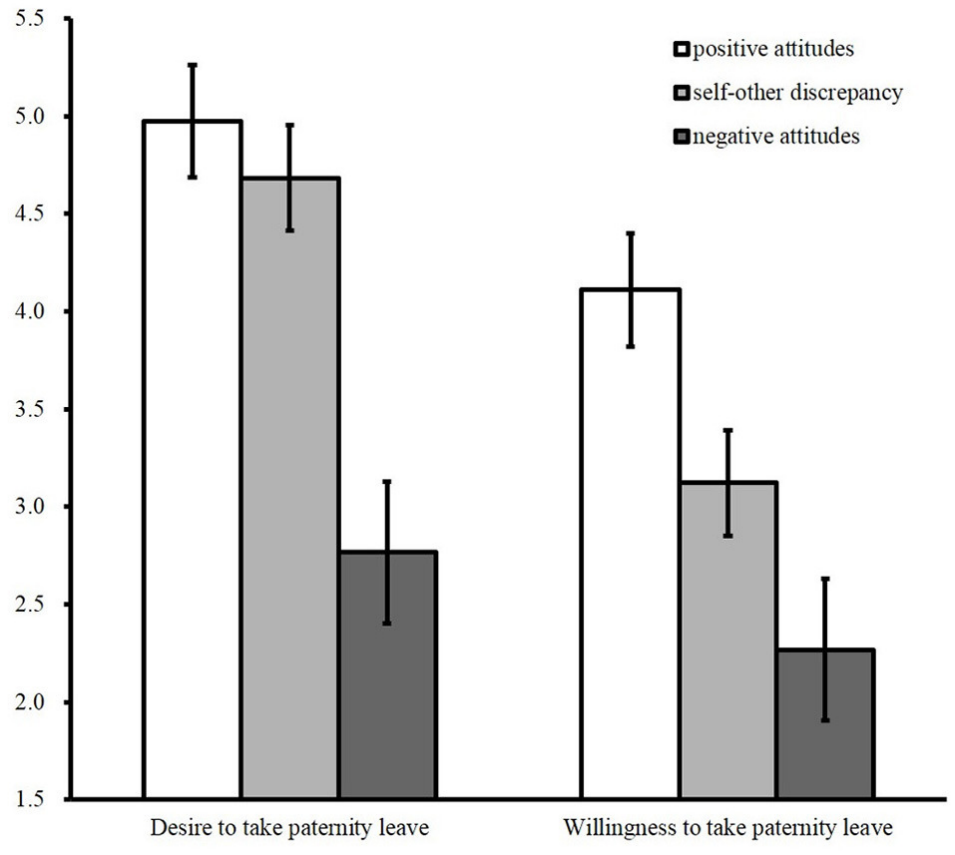
Means of behavioral intentions by group in Study 1 (error bars are 95% confidence intervals).

We performed a 3 (comparison group: positive attitudes vs. self–other discrepancy vs. negative attitudes) × 2 (behavioral intention: desire vs. willingness) mixed-mode analysis of covariance, including age, number of children, length of service, employment status, and traditional gender role orientation in the model as covariates. Traditional gender role orientation was significantly associated with behavioral intentions [*F*_(1, 281)_ = 9.37, *p* < 0.001, η_p_^2^ = 0.03]. None of the effects of covariates except traditional gender role orientation were significant for behavioral intentions (*p*s > 0.23). The results revealed a significant main effect of group, *F*_(2, 281)_ = 44.39, *p* < 0.001, η_p_^2^ = 0.24 and of behavioral intention *F*_(1, 286)_ = 138.60, *p* < 0.001, η_p_^2^ = 0.33, and a significant interaction of these two variables, *F*_(2, 286)_ = 14.59, *p* < 0.001, η_p_^2^ = 0.09. The interaction is displayed in Figure 1.

Multiple comparisons were conducted, using the Bonferroni method. Mean levels of desire in the negative attitudes group were significantly lower than those of the other two groups [*M* = 2.79, *SD* = 1.15; positive attitudes group: *t*_(281)_ = 9.30, *p* < 0.001, *d* = 2.15; self–other discrepancy group: *t*_(281)_ = 8.08, *p* < 0.001, *d* = 1.86], whereas no significant differences existed between the positive attitudes group (*M* = 4.97, *SD* = 1.52) and the self–other discrepancy group [*M* = 4.67, *SD* = 1.55, *t*_(281)_ = 1.52, *p* = 0.39, *d* = 0.39]. Multiple comparisons also showed that the mean level of willingness in the negative attitudes group (*M* = 2.24, *SD* = 1.14) was significantly lower than that in the other two groups [vs. positive attitudes group: *t*_(281)_ = 7.48, *p* < 0.001, *d* = 1.73; vs. self–other discrepancy group: *t*_(281)_ = 3.57, *p* < 0.001, *d* = 0.82]. Furthermore, the mean level of willingness in the self–other discrepancy group (*M* = 3.13, *SD* = 1.62) was significantly lower than that in the positive attitudes group [*M* = 4.11, *SD* = 1.68, *t*_(281)_ = 4.74, *p* < 0.001, *d* = 0.91]. Among all three groups, the divergence between desire and willingness to take paternity leave was largest in the self–other discrepancy group [*t*_(112)_ = 10.37, *p* < 0.001, *d* = 0.97]. Importantly, the mean levels of desire and willingness in the self–other discrepancy group were distributed across the midpoint, and both intentions showed significant differences from the midpoint [desire: *t*_(112)_ = 5.18, *p* < 0.001, *d* = 0.68; willingness: *t*_(112)_ = −5.29, *p* < 0.001, *d* = 0.69]. That is, the average score for desire indicated “I slightly want to take paternity leave,” whereas, that of willingness indicated “I probably would not take paternity leave.” Therefore, Hypothesis 2 was supported.

### Discussion

Study 1 demonstrated that male employees overestimated other men's negative attitudes toward paternity leave. In addition, male employees who mistakenly believed that other men had negative attitudes toward paternity leave were less willing to use paternity leave, although their own attitudes toward taking paternity leave were positive. These results support the hypothesis that perpetual low rate of paternity leave in Japan is induced by pluralistic ignorance.

However, these results may merely reflect social desirability rather than the impact of pluralistic ignorance. Specifically, since people's need to be socially desirable often distorts their responses on self-report measures (Fishman, 1965), it is possible that participants consciously or unconsciously rate the paternity leave more favorably than others to maintain their positive self-images. To eliminate this alternative interpretation of the results, we conducted an additional investigation and attempted to provide more rigorous examination of the findings in Study 2.

## Study 2

Study 2 aimed to examine the association between pluralistic ignorance and the perpetually low rates of taking paternity leave in Japan more directly by controlling for the effect of social desirability (Hines, 2002; Carey et al., 2006; Prince and Carey, 2010).

### Materials and methods

#### Participants

Participants who met the same criteria as those in Study 1 were recruited through the same crowdsourcing service and given the same monetary incentive. Participants completed an online questionnaire in April and May 2017. Those who responded incorrectly to the attention check question were eliminated from the sample, and a total of 425 male participants were included in the final analysis (*M*_age_ = 40.60, *SD* = 5.59, Range = 20–49). Of the 425 participants, 97.9% identified as regular employees. The average length of employment in the current workplace was 13.65 years (*SD* = 7.40), and the average number of children was 1.64 (*SD* = 0.97).

#### Measures

##### Attitudes toward paternity leave

Participants were asked to indicate their own attitudes toward paternity leave and to estimate the average attitudes of men aged 20–49 years, using the same 5 items and 6-point scale used in Study 1. Both scales showed sufficient internal reliabilities (private attitude: Cronbach's alpha of 0.95 and McDonald's omega of 0.95, 95%CI [0.94, 0.96]; estimation of others' attitudes: Cronbach's alpha of 0.95 and McDonald's omega of 0.95, 95%CI [0.94, 0.96]). We computed the average of all private attitude scores to create a single private attitude score, and similarly averaged the others' attitudes item scores to produce an others' attitudes score. The average scores were normally distributed (private attitudes: skewness = −0.45, kurtosis = −0.34; estimation of others' attitudes: skewness = 0.21, kurtosis = −0.10), suggesting that parametric methods would be appropriate for data analysis.

##### Behavioral intention to take paternity leave

Participants were asked to indicate their desire and willingness to take paternity leave on a 7-point scale with the identical items used in study 1. Both responses were normally distributed (desire: skewness = −0.25, kurtosis = −0.74; willingness: skewness = 0.27, kurtosis = −0.93).

##### Control variables.

*Traditional gender role orientation*. Traditional gender role orientation was measured using the same four items from Study 1. Cronbach's alpha was 0.90 and McDonald's omega was 0.90, 95%CI [0.89, 0.92].

*Demographic variables*. Participants' age, number of children, length of service, and employment status were collected as in Study 1.

*Social desirability*. Participants were asked to complete the short form of the Marlowe-Crowne Social Desirability Scale (Reynolds, 1982). This scale consists of 13 items with a true/false response scale (e.g., “*It is sometimes hard for me to go on with my work if I am not encouraged*”). We dropped four items that had low reliability in our sample: “*I'm always willing to admit it when I make a mistake”, “I am always courteous, even to people who are disagreeable”, “I have never been irked when people expressed ideas very different from my own” and “I have never deliberately said something that hurt someone's feelings”*). We computed the total number of “true” answers across the final nine items to create a social desirability score. The scale achieved a Cronbach's alpha of 0.68 and McDonald's omega of 0.68, 95%CI [0.63, 0.73], with higher scores reflecting higher levels of social desirability.

### Results

The means, standard deviations, and correlations of the variables are listed in Table 2. There was small but significant correlation between social desirability and private attitude toward paternity leave. When constructs are examined with a common method (e.g., self-report measure), significant correlations between measures are likely (Podsakoff et al., 2003). To rule out the potential impact of social desirability on the measures, we included the social desirability score as covariate to the model in the subsequent analyses.

**Table 2:** Descriptive statistics and zero-order correlations in Study 2.

	***M***	***SD***	**1**	**2**	**3**	**4**	**5**	**6**	**7**	**8**	**9**	**10**
1. Age	40.60	5.59	–									
2. Number of children	1.64	0.97	0.09^+^	–								
3. Employment status	–	–	0.08^+^	0.05	–							
4. Length of service	13.65	7.40	0.52^**^	0.04	−0.16^**^	–						
5. Traditional gender role orientation	3.40	1.45	0.06	0.08	−0.06	0.08^+^	–					
6. Social desirability	4.27	2.34	−0.10^*^	−0.06	0.03	−0.10^*^	0.16^**^	−				
7. Private attitudes	4.31	1.22	−0.09^+^	−0.02	0.00	−0.15^**^	−0.39^**^	−0.11^*^	–			
8. Estimation of others' attitudes	3.33	1.03	0.04	0.06	−0.05	0.00	−0.12^**^	−0.02	0.35^**^	–		
9. Behavioral intention (desire)	4.48	1.70	−0.08^+^	−0.06	−0.02	−0.15^**^	−0.30^**^	−0.01	0.73^**^	0.31^**^	–	
10. Behavioral intention (willingness)	3.67	1.78	0.00	−0.03	0.04	−0.08^+^	−0.23^**^	−0.04	0.66^**^	0.43^**^	0.76^**^	–

*N = 425; employment status (1 = regular employee, 2 = contract employee and temporary worker); social desirability was calculated with nine items*.

** *p < 0.01*,

* *p < 0.05*,

+ *p < 0.10*.

#### Occurrence of pluralistic ignorance

To assess the self–other discrepancy in attitudes toward paternity leave, we conducted analysis of covariance, including age, number of children, length of service, employment status, traditional gender role orientation, and social desirability in the model as covariates. Traditional gender role orientation was significantly associated with attitudes [*F*_(1, 418)_ = 47.42, *p* < 0.001, η_p_^2^ = 0.10]. None of the effects of covariates except traditional gender role orientation were significant (*p*s > 0.055). Estimations of others' attitudes toward paternity leave (*M* = 3.33, *SD* = 1.03) were significantly lower than the participants' actual attitudes [*M* = 4.31, *SD* = 1.22; *F*_(1, 424)_ = 246.01, *p* < 0.001, η_*p*_^2^ = 0.37]. Again, participants estimated the attitudes of their peers toward paternity leave to be more negative than they actually were. This is additional clear support for Hypothesis 1. Furthermore, private attitudes and the estimations of others' attitudes significantly diverged across the midpoint [private attitude: *t*_(418)_ = 13.63, *p* < 0.001, *d* = 0.97; estimation of others' attitudes: *t*_(418)_ = −3.32, *p* < 0.01, *d* = 0.22].

#### Influence of perceived self–other discrepancy on behavioral intention

**Figure 2:**
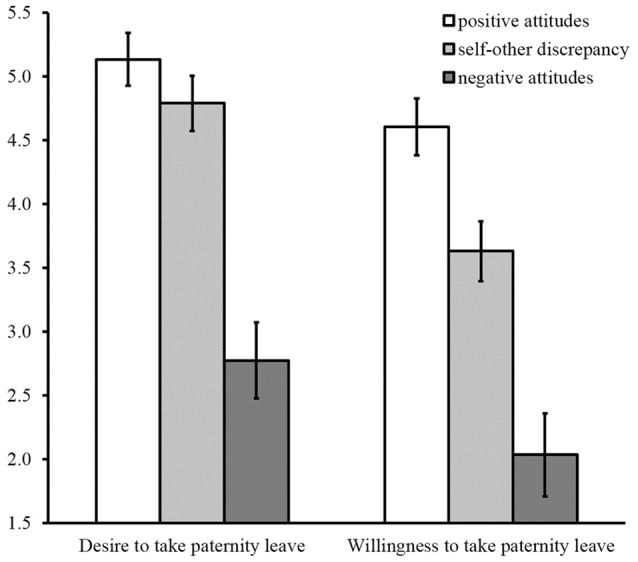
Means of behavioral intentions by group in Study 2 (error bars are 95% confidence intervals).

We performed a 3 (comparison group: positive attitudes vs. self–other discrepancy vs. negative attitudes) × 2 (behavioral intention: desire vs. willingness) mixed-mode analysis of covariance, including age, number of children, length of service, employment status, traditional gender role orientation, and social desirability in the model as covariates. Traditional gender role orientation was significantly associated with behavioral intentions [*F*_(1, 402)_ = 11.87, *p* < 0.001, η_p_^2^ = 0.03]. None of the covariates except traditional gender role orientation were significant for behavioral intentions (*p*s >.12). The main effects of group, *F*_(2, 402)_ = 95.52, *p* < 0.001, η_p_^2^ = 0.32 and of behavioral intention *F*_(1, 408)_ = 180.36, *p* < 0.001, η_p_^2^ = 0.31, were significant, as was the interaction between these variables, *F*_(2, 408)_ = 13.32, *p* < 0.001, η_p_^2^ = 0.06. The interaction is displayed in Figure 2.

Participants were categorized into four groups as in Study 1 (positive attitudes group: *N* = 170, self–other discrepancy group: *N* = 157, negative-positive attitudes group: *N* = 14, negative attitudes group: *N* = 84). As in Study 1, participants in the negative-positive attitudes group were excluded from the subsequent analysis because of the small sample size. Multiple comparisons using the Bonferroni method confirmed that the mean levels of desire in the negative attitudes group were significantly lower than those in the other two groups [*M* = 2.77, *SD* = 1.22; positive attitudes group: *t*_(402)_ = 12.59, *p* < 0.001, *d* = 2.46; self–other discrepancy group: *t*_(402)_ = 10.65, *p* < 0.001, *d* = 2.10]. No significant differences emerged between the positive attitudes group (*M* = 5.13, *SD* = 1.44) and the self–other discrepancy group [*M* = 4.79, *SD* = 1.42, *t*_(402)_ = 2.27, *p* = 0.07, *d* = 0.48].

Multiple comparisons were conducted, using the Bonferroni method. The mean level of willingness in the negative attitudes group (*M* = 2.03, *SD* = 0.95) was significantly lower than that in the other two groups [vs. positive attitudes group: *t*_(402)_ = 12.65, *p* < 0.001, *d* = 2.47; vs. self–other discrepancy group: *t*_(402)_ = 7.77, *p* < 0.001, *d* = 1.53]. Furthermore, the mean level of willingness in the self–other discrepancy group (*M* = 3.63, *SD* = 1.56) was significantly lower than that in the positive attitudes group [*M* = 4.60, *SD* = 1.62, *t*_(402)_ = 5.92, *p* < 0.001, *d* = 0.94]. Among all three groups, the divergence between desire and willingness to take paternity leave was largest in the self–other discrepancy group [*t*_[150]_ = 11.24, *p* < 0.001, *d* = 0.80]. Importantly, the mean levels of desire and willingness in the self–other discrepancy group was distributed across the midpoint, and both intentions showed significant differences from the midpoint [desire: *t*_(150)_ = 7.14, *p* < 0.001, *d* = 0.83; willingness: *t*_(150)_ = −2.77, *p* < 0.05, *d* = 0.31]. These results indicate support for Hypothesis 2.

### Discussion

The results of Study 2 completely replicated the findings of Study 1, even after controlling for social desirability. Again, male employees overestimated other males' negative attitudes toward paternity leave, and this misperception impeded their behavioral intentions to take paternity leave. These results support the robustness of the interpretation that perpetually low rates of paternity leave in Japan could reflect pluralistic ignorance.

A potential concern in Study 2 is the reliability of the social desirability scale. We eliminated some items to obtain an acceptable reliability coefficient (Hinkin, 1995). We adopted Reynolds' (1982) scale to be consistent with previous studies of pluralistic ignorance; however, several social desirability scales have been developed, including the Japanese version of the Balanced Inventory of Desirable Responding (BIDR; Paulhus, 1991), with demonstrated adequate reliability and validity (Tani, 2008). Therefore, future research might consider use of another social desirability scale.

## General discussion

In the current study, we sought to determine whether Japanese male employees overestimated other male employees' negative attitudes toward paternity leave, and whether this misperception can lead them not to follow through with intentions to use paternity leave. Although most of male employees in Japan aspire to take paternity leave when they have a child, few of them actually make the most of the paternity leave policy (NetMile Research, 2009; Japanese Ministry of Health, 2015). Previous studies indicate that organizational climate has a critical impact on the use of paternity leave (e.g., Blair-Loy and Wharton, 2002). Here, assuming that most of the male employees now have positive attitudes toward paternity leave, how is this gender-unequal social practice perpetuated in Japan? To further understand this inconsistency between attitudes and behaviors, we conducted two web-based questionnaires among Japanese male employees in their 20 to 40 s.

The results of Study 1 demonstrated that male employees overestimated other men's negative attitudes toward paternity leave. Moreover, those who had positive attitudes and estimated others to have negative attitudes (i.e., the self–other discrepancy group) were less willing to take paternity leave than were those who had positive attitudes and estimated that others have positive attitudes as well (i.e., the positive attitudes group). However, there was no significant difference in the two groups' desire to take paternity leave. That is, although individual men had positive attitudes toward taking paternity leave, their inaccurate assumption that other men have negative attitudes toward taking such leave restrains them from behaving in accordance with their private beliefs. Instead, they responded according to the perceived group norm (i.e., relinquishing paternity leave). This tendency is consistent with the literature on pluralistic ignorance (e.g., Prentice and Miller, 1996). Moreover, Study 2 replicated these results and further indicated that they could not be explained by the participants' needs to be appear socially desirable (Reynolds, 1982). Taken together, our findings suggest that the social issue of paternity leave in Japan is characterized by pluralistic ignorance.

Why do male employees fail to perceive others' beliefs accurately? This question might be answered from several perspectives. From the societal-level perspective, one possible explanation is “conservative lag” or “cultural lag,” which is a form of pluralistic ignorance (Breed and Ktsanes, 1961; Fields and Schuman, 1976). The terms refer to a phenomenon in which a “public norm that once had private support continues in place despite having lost private support” (see Miller and Prentice, 1994; Vandello and Cohen, 2004 for a review). The concept was established in the late 1960s and early 1970s in the historical context of considerable concern in U.S. society regarding racial segregation. At that time, many Caucasian Americans were uncomfortable with the idea of segregation but overestimated peers' endorsement of segregation (Breed and Ktsanes, 1961; O'Gorman, 1975; Fields and Schuman, 1976). That is, segregation continued even after losing majority support because of people's misperception that the majority of their peers supported segregation. Thus, changes in social norms often lag behind changes in individuals' attitudes. Only a few topics of pluralistic ignorance (e.g., racial discrimination toward African Americans in the late 1960s and early 1970s, the honor culture in the southern U.S.) have been attributed to conservative lag.

The ongoing low rate of using paternity leave in Japan may be another phenomenon explained by conservative lag. Indeed, the majority of individuals in Japan espoused traditional gender role norms in the past (Japanese Cabinet Office, 2002). However, these values have shifted over time, while public behaviors have not. For this reason, male employees believed that changes in their own attitudes were not shared by their colleagues. Consequently, under the mistaken assumption that the majority of their peers continue to support the conservative view, the majority of the male employees acquiesced to the prevailing social practice while no longer privately supporting it. From an individual-level perspective, people's misperceptions might be partially sustained by attribution bias, i.e., the tendency to overemphasize internal dispositions to explain others' behaviors in a given situation rather than considering the situational factors (e.g., Jones and Nisbett, 1971). Moreover, others' behaviors are often interpreted as approach-motivated despite the recognition that one's own identical behaviors are avoidance-motivated (Miller and Nelson, 2002). That is, when male employees observe that their colleagues do not take paternity leave, they may assume that their colleagues hold a belief that men should not take paternity leave.

Decisions and behaviors by individuals often produce significant unintended consequences for an entire group. When we seek to determine appropriate decisions and behaviors in a given situation, reference to the prevailing social practice and use of a social comparison process (Festinger, 1954) might play a particularly critical role. In the situation of pluralistic ignorance, the assumption that others still cling to conservative beliefs is confirmed and reinforced by observing others' behaviors. At the same time, one's own behavioral conformity might also contribute to convincing other group members to conform to the perceived norm. In this sense, the conviction that the norm has majority support is strengthened through the social comparison process and thus generates widespread conformity, which finally reaches behavioral equilibrium as a self-fulfilling prophecy (Cohen, 2001). Thus, pluralistic ignorance contributes to perpetuating unpopular norms (Vandello and Cohen, 2004).

Empirical findings indicate that males' attitudes toward gender roles have undergone a broad shift from conservative to gender-equal. Recent studies (Lopez-Zafra and Garcia-Retamero, 2012, Spanish sample; Wilde and Diekman, 2005, US and German sample; Yukawa and Hirooka, 2003, Japanese sample) have revealed that beliefs about masculine and feminine characteristics were becoming more similar. However, those studies suggest that the perceived gender stereotype has been weakened through women's acquisition of more masculine characteristics. In contrast, the findings of our studies imply that Japanese men are gaining more “feminine-communal” traits (i.e., traits deeply involved in child caring). Thus, gender equality might be further developed by men's advancing into homes as well as women's advancing into society.

Most existing intervention strategies in Japan have attempted to encourage taking paternity leave by changing men's private attitudes toward paternity leave or by incentivizing companies to support the employees' use of paternity leave policy (e.g., Japanese Cabinet Office, 2015). The current findings indicate an alternative solution—altering erroneous assumptions about other male employees' attitudes, rather than attempting to alter one's own private attitudes. Previous studies have demonstrated that feedback about the self–other discrepancy in perceived group norms could correct misperceptions and reduce conformity to the misperceived norm (Schroeder and Prentice, 1998; Neighbors et al., 2004; Walters and Neighbors, 2005). Therefore, strategies such as sharing information on the actual attitudes of employees in the workplace, or arranging seminars on pluralistic ignorance to promote understanding of the phenomenon could be effective ways to encourage male employees to claim paternity leave. The realization of shared misperception of norms could empower male employees to achieve their aspirations and, consequently contribute to promoting gender equality in Japan.

Findings in the current study should be interpreted with caution for several reasons. First, we cannot determine causality between pluralistic ignorance and behavioral intentions to take paternity leave because our study used a cross-sectional design. Therefore, causality between them should be explored with a more rigorous design in the future. Second, we adopted an operational definition of pluralistic ignorance as the individuals' misperceptions (i.e., self–other discrepancy). Although the data clearly demonstrated that normative misperception suppresses individuals' behavioral intentions, some researchers might argue that future research should consider pluralistic ignorance as a group-level phenomenon. Therefore, future study should seek to use methodological advancements (e.g., multilevel models; Halbesleben et al., 2007) to capture the group process (i.e., interaction between individuals) rather than individuals' erroneous perceptions of perceived norms.

Third, the participants in the current study (i.e., the Yahoo! Crowdsourcing panel) might represent a specific population of Internet users. In addition, we did not examine the relevance of socioeconomic status to our findings. Previous studies (Brandth and Kvande, 2002, Norwegian sample; Lammi-Taskula, 2008, Finnish sample) suggest that socioeconomic variables such as family economic level (including father's income level compared with that of the mother), occupation (“blue-collar” or “white-collar”), sector of employment (public vs. private) and educational level of the couple are relevant to taking paternity leave. Therefore, findings of the current study might be applicable to certain Japanese men of a specific socioeconomic background. Future research to examine the influence of relevant variables on pluralistic ignorance in specific organizations is recommended.

Despite the aforementioned limitations, our findings may benefit both researchers and practitioners. The results are consistent with the existing theoretical framework of pluralistic ignorance and the findings of recent surveys on paternity leave in Japan. The current research provides an important first step in understanding the dynamics of taking paternity leave and in elucidating the factors that could promote male employees to take such leave in Japan. Dispelling pluralistic ignorance on this social issue could possibly lead to behavioral changes in fathers: “I want to, therefore I will.”

## Author contributions

Conceived and designed the survey and wrote the paper: TM and HY. Analyzed the data: TM.

### Conflict of interest statement

The authors declare that the research was conducted in the absence of any commercial or financial relationships that could be construed as a potential conflict of interest.

## Footnotes

^1^The questions were based on the prevalent saying, ‘*Men make houses, women make homes*,’ which refers to Japanese conservative gender role stereotypes. Although the saying seems to comprise two ideas, most Japanese people generally recognize it as a single concept. Therefore, participants were less likely to confuse the items. This assumption is supported by high internal consistencies (i.e., Cronbach's alpha and McDonald's omega).

^2^The range of error (error bars are 95% confidence intervals) of means of behavioral intentions in each group are not so wide as depicted in Figures 1, 2. This fact demonstrates that the effect of the distribution of participants on variability of data is small.
